# Initial experience with the antithrombogenic-coated CARESTO stent for venous sinus stenting for idiopathic intracranial hypertension: A multicenter study

**DOI:** 10.1177/15910199251392958

**Published:** 2025-11-04

**Authors:** Mohammad Almohammad, Gabriel Broocks, Peter B Sporns, Mete Dadak, Bayan Alhaj Moustafa, Ali Khanafer, Tawfik Moher Alsady, Lisa Hekers, Christopher Nimsky, Alexander Grote, Lars Timmermann, Anja Gerstner, Theo Demerath, Joachim Klisch, Donald Lobsien, André Kemmling

**Affiliations:** 1 Department of Neuroradiology, 61061 Philipps-University Marburg, Campus Marburg, Marburg, Germany; 2 Department of Neuroradiology, University Hospital of Giessen and Marburg, Campus Marburg, Marburg, Germany; 3 Department of Neuroradiology, 506874 HELIOS Medical Center, Campus of MSH Medical School Hamburg, Schwerin, Germany; 4 Department of Neuroradiology, 30262 University Hospital Basel, Basel, Switzerland; 5 Department of Radiology and Neuroradiology, 31012 Stadtspital Zürich Triemli, Zürich, Switzerland; 6 Department of Radiology and Neuroradiology, 9177 St Vincenz Hospital Paderborn, Paderborn, Germany; 7 Department of Neuroradiology, 9203 Klinikum Stuttgart Katharinenhospital, Stuttgart, Germany; 8 Department of Radiology and Neuroradiology, 39515 Rhön-Klinikum, Campus Bad Neustadt, Bad Neustadt an der Saale, Germany; 9 Department of Neuroradiology, Faculty of Medicine, Medical Center – University of Freiburg, Freiburg, Germany; 10 Health and Medical University Erfurt & Clinic for Neuroradiology, 62480 HELIOS Hospital Erfurt, Erfurt, Germany; 11 Department of Neuroradiology, 62480 Institute for Diagnostic and Interventional Radiology and Neuroradiology, Chemnitz Clinic, Chemnitz, Germany; 12 Medical Campus Chemnitz, Technical University Dresden, Chemnitz, Germany

**Keywords:** CARESTO, venous sinus stenting, idiopathic intracranial hypertension

## Abstract

**Objectives:**

To evaluate the feasibility, safety, and short-term efficacy of the antithrombogenic-coated CARESTO stent for venous sinus stenting (VSS) in idiopathic intracranial hypertension (IIH).

**Methods:**

This retrospective multicenter study included IIH patients with venous sinus stenosis fulfilling the modified Dandy criteria, elevated lumbar puncture opening pressure, and a trans-stenotic pressure gradient ≥ 7 mmHg. Clinical endpoints were improvement of headache, papilledema, and visual function. Imaging endpoints included stent patency, venous sinus diameter, and restenosis. Physiological endpoints were the reduction of the trans-stenotic pressure gradient and lumbar puncture opening pressure. Safety was assessed by recording peri- and postprocedural complications.

**Results:**

Between January 2023 and August 2025, 16 IIH patients underwent VSS with the CARESTO stent at four centers. Mean age was 33.9 ± 6.9 years; 75% were female. Stent deployment was technically successful in all cases. The median trans-stenotic gradient fell from 18 to 3 mmHg and lumbar puncture pressure from 34 to 16.5 cmH₂O at 3 months, with sinus diameter increasing from 2.0 to 7.0 mm. All patients improved clinically, 81.3% within 24 hours. At 3 months, all stents were patent without restenosis, complications, or mortality.

**Conclusion:**

VSS with the antithrombogenic-coated CARESTO stent appears feasible, safe, and effective, providing consistent short-term clinical, hemodynamic, and physiological benefits in IIH. Further prospective studies in larger cohorts are warranted and may open the door to performing VSS under single antiplatelet therapy.

## Introduction

Idiopathic intracranial hypertension (IIH) is a debilitating condition characterized by elevated intracranial pressure, most often affecting young women, and frequently leading to chronic headache, papilledema, and progressive visual loss.^1^ An elevation in venous sinus pressure due to dural sinus stenosis (DSS) is increasingly recognized as a key mechanism in the pathogenesis of intracranial hypertension. Such stenoses are frequently observed in patients with IHH, particularly within the transverse sinus.^2^

Conservative management, including weight reduction, diuretics, and serial lumbar punctures, remains the first-line therapy. In refractory cases or those with progressive visual decline, surgical interventions such as cerebrospinal fluid (CSF) shunting or optic nerve sheath fenestration are traditionally considered. Recently, venous sinus stenting (VSS) has emerged as an established option for selected patients with significant sinus stenosis and trans-stenotic pressure gradient, demonstrating high technical success, intracranial pressure reduction, and symptomatic improvement.^3–6^ Nevertheless, important anatomical and technical challenges remain. Conventional carotid stents, while providing sufficient radial force, are often too stiff for navigation through the tortuous anatomy of the dural venous sinuses and may compromise precise deployment and optimal wall apposition.^7^ In contrast, more flexible intracranial stents provide superior navigability but often lack the radial strength and the available diameters and lengths required for effective sinus reconstruction. These limitations underscore the need for devices specifically engineered for the unique anatomical demands of the dural venous sinuses.^8^

New generations of stent systems, many featuring surface-modified designs, are emerging—enhancing biocompatibility and reducing thrombogenicity, thereby paving the way for safer and more versatile neuroendovascular therapies and introducing new catheterization techniques.^9–12^

The CARESTO (Acandis, Germany, CE-marked for the treatment of carotid artery stenosis involving the common and internal carotid arteries, including the carotid bifurcationstent) is a new hybrid vascular implant that combines the radial strength of conventional carotid stents with the flexibility and deliverability of braided intracranial designs. Its low-profile delivery system facilitates safe navigation through venous sinus anatomy and accurate placement, while the availability of longer lengths allows coverage of extended stenotic segments. Furthermore, its fibrin/heparin antithrombogenic coating (Heal) is designed to mitigate the risk of thromboembolic complications.^8^ Although its use remains off-label, this device has recently been adopted for VSS in several high-volume neurovascular centers; however, its use in this indication has not yet been systematically studied. Therefore, this study represents the first multicenter clinical evaluation of an antithrombogenic-coated hybrid stent for VSS in IIH, aiming to provide real-world evidence for a novel device concept specifically tailored to the challenges of tortuous vessel anatomy. While the routine use of single antiplatelet therapy in VSS cannot yet be justified, the antithrombogenic coating of this device may support future investigations into whether safe implantation under monotherapy is feasible.

## Methods

### Study design

A retrospective multicenter study. Ethical approval was obtained from the central ethics committee of the coordinating institution (Philipps University Marburg; IRB number 24-180 RS), which waived the requirement for individual patient consent due to the retrospective design and the use of fully anonymized data. Due to the retrospective and anonymized nature of the data, no additional IRB approvals were required at the collaborating centers. All data were collected and analyzed in a fully anonymized manner in compliance with institutional and national data protection regulations.

### Study participants

Patients were eligible for inclusion if they had a confirmed diagnosis of IIH according to the modified Dandy criteria, including elevated lumbar puncture opening pressure. Typical clinical features included headache, papilledema, and/or visual disturbances. Only patients with a documented trans-stenotic venous pressure gradient of ≥ 7 mmHg were included. Radiological confirmation of venous sinus stenosis was required and had to be demonstrated by at least one imaging modality, including magnetic resonance venography (MRV), computed tomography venography (CTV), or digital subtraction angiography (DSA). Both unilateral and bilateral transverse or sigmoid sinus stenoses were eligible. No additional exclusion criteria were applied.

### Endovascular procedures

**Figure 1.**
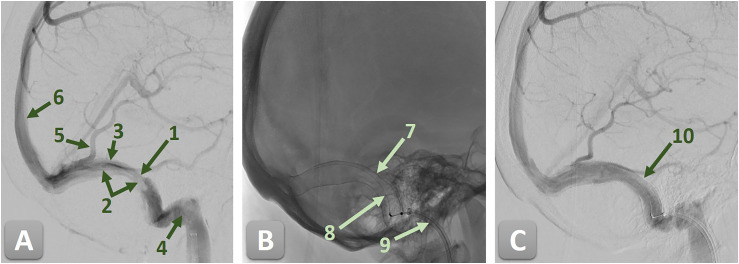
Digital subtraction angiography (DSA) of the right sigmoid sinus (SS) demonstrating a high-grade stenosis secondary to an old, organized sinus thrombosis, before and after endovascular treatment with a CARESTO stent. 1—high-grade stenosis of the right SS; 2—organized sinus thrombosis; 3—SS; 4—jugular bulb; 5—Vein of Labbé; 6—superior sagittal sinus; 7—CARESTO stent; 8—stent pusher; 9—guiding catheter; and 10—completely resolved stenosis. (A) Pre-treatment angiogram showing a high-grade stenosis of the right SS due to an old, organized thrombosis. (B) Unsubtracted angiogram after implantation, depicting the CARESTO stent in situ with the stent pusher still in place. (C) Post-treatment angiogram confirming successful reconstruction of the SS with complete resolution of the stenosis and restoration of normal venous outflow.

**Figure 2.**
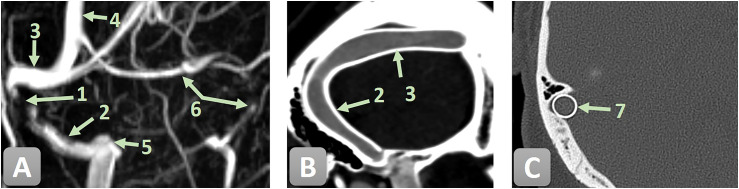
Pre-interventional MRV and three-month post-interventional CT/CTV follow-up of the venous sinuses illustrating a high-grade stenosis of the right SS before and after endovascular treatment with a CARESTO stent. 1—high-grade stenosis of the right SS; 2—SS; 3—transverse sinus; 4—superior sagittal sinus; 5 —jugular bulb; 6—hypoplastic left transverse and sigmoid sinus; and 6—CARESTO stent. (A) Pre-treatment MR venogram demonstrating a high-grade stenosis of the right SS. (B) Three-month follow-up CTV showing the CARESTO stent in place with no residual stenosis. (C) Three-month follow-up CT depicting a cross-sectional view of the CARESTO stent within the previously stenotic segment, confirming complete luminal reconstruction and adequate radial force.

MRV: magnetic resonance venography; CT: computed tomography; CTV: computed tomography venography; SS: sigmoid sinus.

**Figure 3.**
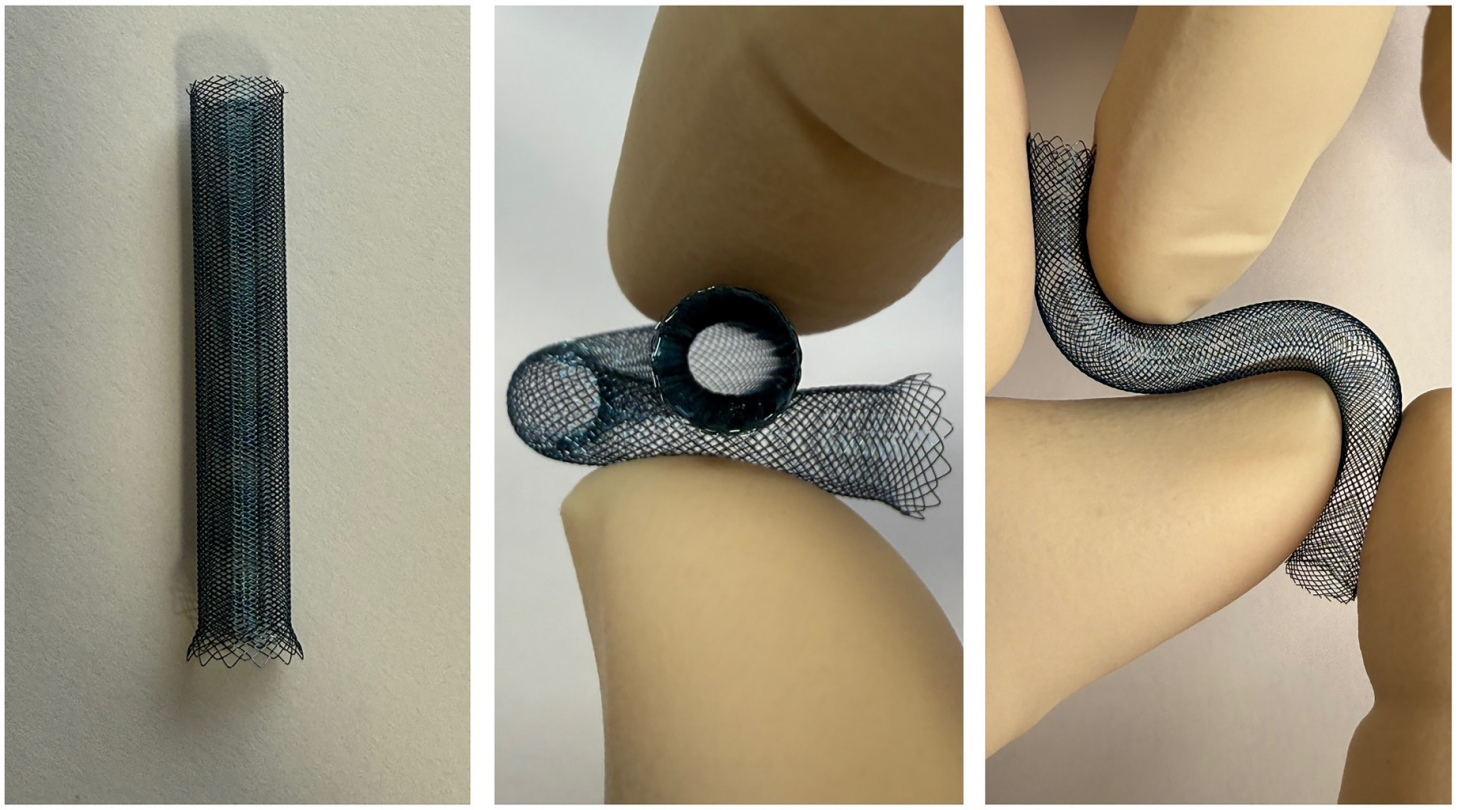
Representative images of a 6 × 50 mm antithrombogenic-coated CARESTO stent demonstrating its hybrid architecture, flexibility, and adaptability for vessel reconstruction in tortuous anatomy.

All procedures were performed under general anesthesia using a biplane angiographic system. Vascular access was obtained via an 8F femoral venous sheath and a 5F femoral arterial sheath. A diagnostic catheter was positioned in the internal carotid artery (ICA) for contrast injections during the intervention, while a bi- or triaxial venous access system was advanced under fluoroscopic guidance according to individual anatomical and procedural requirements. Prior to stent implantation, percutaneous transluminal angioplasty (PTA) of the DSS was performed, followed by implantation of the CARESTO stent using the recommended NeuroSlider 0.052″ delivery catheter (Acandis, Germany). In cases of suboptimal wall apposition, post-dilatation with PTA was carried out to achieve optimal stent expansion and wall adaptation (Figures 1 to 3). Stent diameter was typically selected 1–2 mm larger than the distal venous sinus diameter to ensure optimal wall apposition, and stent length was chosen to fully cover the stenotic segment with an additional 5–10 mm safety margin on both ends.

### Antithrombogenic regimen

At the beginning of the procedure, all patients received an intravenous bolus of 4000 IU heparin. Antiplatelet preparation consisted of acetylsalicylic acid (ASA) 100 mg and clopidogrel 75 mg once daily for 5 days prior to the intervention. Two hours after stent implantation, all patients underwent a CT protocol including CTV to exclude hemorrhagic or thrombotic complications. Platelet inhibition was monitored using the VerifyNow^®^ system (Werfen, Spain), and the antiplatelet regimen was adjusted if necessary. Dual antiplatelet therapy (DAPT) with ASA and clopidogrel was maintained for three months, after which patients were continued on lifelong ASA 100 mg once daily as secondary prevention, provided that in-stent stenosis had been excluded on three-month follow-up CTV.

### Clinical endpoints

Clinical outcomes were evaluated based on improvement of headache, visual function, and papilledema. Clinical examinations were performed at baseline (prior to initiation of treatment), on the day before the intervention, on the day after the intervention, and at the three-month follow-up. Procedural safety was assessed by documenting any procedure-related complications, including in-stent thrombosis, restenosis, vessel perforation, hemorrhagic events, and secondary venous occlusions.

### Imaging endpoints

Imaging endpoints included assessment of stent patency, changes in sinus diameter at the stenotic segment, and detection of restenosis. Procedural success was defined as restoration of physiological venous outflow with < 50% residual stenosis. Standardized early post-procedural imaging was performed to evaluate stent patency and exclude complications, consisting of non-contrast CT and CTV at 2 and 48 hours after the intervention. At 3 months, follow-up vascular imaging with CTV was obtained in all patients. Imaging analysis focused on stent patency, presence of in-stent stenosis, and detection of thrombotic or other adverse events.

$$\%stenosis=\left[1-\frac{diameter\;of\;stenosis}{diameter\;of\;parent\;vessel}\right]\times 100$$

### Physiological endpoints

Physiological efficacy was determined by hemodynamic and pressure measurements. Procedural success was defined as a reduction of the trans-stenotic venous pressure gradient to < 7 mmHg with restoration of physiological venous outflow at the end of the procedure, together with a decrease of the lumbar puncture opening pressure to ≤ 25 cmH₂O at the three-month follow-up.

### Data collection

Demographic, clinical, imaging, procedural, and follow-up data were retrospectively collected and managed in Microsoft Excel. Procedural information, including materials, techniques, and complications, was systematically documented. Follow-up imaging was evaluated via the institutional PACS. Data integrity was ensured through standardized documentation and cross-validation.

### Data analysis

Continuous variables were expressed as mean ± SD or median (IQR), and categorical variables as counts and percentages. Group comparisons used *t*-tests or Mann–Whitney *U* tests for continuous data and chi-square or Fisher's exact tests for categorical data. Statistical significance was defined as *p* < 0.05. Analyses were performed on complete cases using Jamovi v27.0 (IBM, Armonk, NY).

## Results

### Demographic, clinical, and imaging characteristics

Between January 2023 to August 2025, 16 patients with IIH underwent VSS with the CARESTO stent across four participating neurovascular centers. No patients were excluded. The mean age was 33.9 ± 6.9 years (range, 22–47 years), and 12 patients (75%) were female. All patients fulfilled the modified Dandy criteria and demonstrated a trans-stenotic venous pressure gradient of ≥ 7 mmHg prior to intervention. The transverse sinus was the affected site in all cases (nine right-sided, 56.3%). The median pre-interventional trans-stenotic pressure gradient was 18 mmHg (14–22). The median lumbar puncture opening pressure was 31 cmH₂O (29–34) before intervention (Table 1).

**Table 1.** Demographic, clinical, and imaging characteristics.

Parameters	*N* (%)/mean ± SD/ median (IQR)
Age (years)	33.9 ± 6.9
Gender, female	12 (75%)
Location of stenosis: transverse sinus	16 (100%)
Side of stenosis: right	9 (56.3%)
Pre-interventional trans-stenotic gradient (mmHg)	18 (14–22)
Pre-interventional lumbar opening pressure (cmH_2_O)	34 (31–41)
Diameter at stenosis prior to intervention (mm)	2.0 (2.0–3.0)
Distal sinus diameter prior to intervention (mm)	7.5 (6.0–8.0)
Proximal sinus diameter prior to intervention (mm)	9.0 (8.0–9.0)

IQR: interquartile range; SD: standard deviation.

### Clinical endpoints

All 16 patients demonstrated clinical improvement following VSS. Headache, papilledema, and visual disturbances improved in every case. The majority of patients (13/16, 81.3%) reported early clinical improvement within 24 hours after the intervention, and all patients were free of IIH-related symptoms at the three-month follow-up. No deterioration of IIH-related symptoms or mortality was observed during follow-up (Tabel 2).

**Table 2.** Clinical, imaging, and procedural endpoints.

Parameters	*N* (%)/mean ± SD/ median (IQR)
Early clinical improvement within 24 hours	13/16 (81.3%)
Symptom resolution at 3 months	16/16 (100%)
Post-interventional trans-stenotic gradient (mmHg)	3 (2–3)
Lumbar opening pressure at 3 months (cmH_2_O)	16.5 (13–18)
Diameter at stenosis after intervention (mm)	7.0 (6.0–8.0)
Mean deployed stent diameter (mm)	8.9 ± 0.7
Mean stent length (mm)	59.4 ± 3.1

IQR: interquartile range; SD: standard deviation.

### Imaging endpoints

Early post-procedural CT and CTV at 2 and 48 hours confirmed stent patency in all patients, without hemorrhage, thrombosis, or venous occlusion. At 3 months, CTV demonstrated persistent patency in all 16 cases, with no in-stent stenosis or thrombotic complications. The median diameter at the stenotic site increased from 2.0 mm (2–3) pre-intervention to 7.0 mm (6–8) post-intervention, while distal and proximal diameters measured 7.5 mm (6–8) and 9.0 mm (8–9), respectively. Residual stenosis remained < 50% in all cases, meeting the imaging definition of procedural success. Single-stent reconstruction was sufficient in all 16 patients; no telescoping or overlapping stents were required. The mean deployed stent diameter was 8.9 ± 0.7 mm, with a mean length of 59.4 ± 3.1 mm (Table 2). The braided hybrid stent design provided sufficient lumen stability and flexibility, allowing uncomplicated catheterization and contrast passage through the reconstructed sinus.

### Physiological endpoints

The median pre-interventional trans-stenotic venous pressure gradient decreased from 18 mmHg (14–22) to 3 mmHg (2–3) after VSS. The median lumbar puncture opening pressure declined from 34 cmH₂O (31–41) prior to the procedure to 16.5 cmH₂O (13–18) at three-month follow-up (Table 2).

### Procedural safety

No major periprocedural complications occurred. Specifically, there were no cases of in-stent thrombosis, relevant restenosis, vessel perforation, hemorrhagic events, air embolism, secondary venous occlusions, or mortality. The intervention was well tolerated across all centers, demonstrating a consistently favorable safety profile.

## Discussion

This multicenter retrospective study suggests that VSS for IIH using the antithrombogenic-coated CARESTO stent is technically feasible and appears to be associated with favorable short-term clinical, imaging, and physiological outcomes. All patients demonstrated clinical improvement with resolution of IIH-related symptoms during follow-up, and no major periprocedural complications were observed. Importantly, the absence of in-stent thrombosis, restenosis, or venous occlusion may reflect a potential benefit of the stent's antithrombogenic surface coating, while its hybrid design likely contributes to improved navigability and sustained venous patency.

Stent deployment was technically successful in every case, and procedural efficacy was reflected by a marked reduction in the trans-stenotic pressure gradient (from 18 to 3 mmHg) and a significant decrease in lumbar puncture opening pressure (from 34 to 16.5 cmH₂O). Imaging follow-up confirmed stable stent patency, expansion of the stenotic segment diameter (2–7 mm), and absence of restenosis. Clinically, the majority of patients (81.3%) experienced symptomatic relief within 24 hours, and all were symptom-free at 3 months. Taken together, these results underscore the consistency of the clinical and hemodynamic improvements achieved with VSS in this selected patient population.

Our findings align with previous large series reporting the safety and efficacy of VSS in IIH. Labeyrie et al.^13^ analyzed 200 patients treated mainly with the Carotid Wallstent and observed a 79% primary symptom resolution rate, 10% recurrence, and no mortality over a median follow-up of 2.2 years. In contrast, Bilgin et al.^7^ directly compared the Zilver and Carotid Wallstent in 76 patients and demonstrated that stent type may influence outcomes. Specifically, Zilver stents—longer and more flexible—were associated with significantly shorter procedure times, greater reduction in pressure gradients, and higher rates of headache and tinnitus resolution.

Compared with these studies, our series with the antithrombogenic-coated CARESTO stent demonstrated 100% technical success, universal short-term symptom resolution, and no thrombotic, hemorrhagic, or restenosis events during follow-up. While these encouraging outcomes may reflect advantages of the stent's hybrid design—combining improved navigability, sufficient radial force—the small cohort size and limited follow-up restrict definitive conclusions. Larger prospective and comparative studies are needed to determine whether coated hybrid stents confer additional benefits over conventional devices.

In previous series using non-coated stents for venous sinus stenting, in-stent thrombosis has been reported in ∼2%–15% of cases.^14^ In contrast, no thrombotic or restenotic events occurred in our cohort either peri-procedurally or at three-month follow-up. While this may reflect the potential benefit of the stent's antithrombogenic coating, the small sample size precludes definitive conclusions.

Nevertheless, this initial multicenter experience suggests that the CARESTO stent may represent a safe and effective option for VSS in carefully selected IIH patients, potentially informing future treatment strategies, and may provide a rationale for evaluating single antiplatelet therapy in this context.

### Limitations

This study has important limitations. Its retrospective design, small sample size, and short follow-up restrict the generalizability of the findings and preclude definitive conclusions on long-term safety and efficacy. The absence of a control group limits direct comparison with other stents or with CSF shunting. Additionally, multicenter variability in patient selection and technique may have influenced outcomes. Future prospective studies with larger cohorts and longer follow-up are warranted to validate these results, to compare coated with uncoated stents, and to better define the role of VSS in the treatment algorithm of IIH.

## Conclusion

This first multicenter systematic clinical evaluation of the antithrombogenic-coated CARESTO stent for VSS in IIH indicates that the implantation of the device appears technically feasible, safe, and effective in the short term, providing consistent improvements in symptoms, trans-stenotic pressure gradients, and venous sinus caliber. The absence of thrombotic, hemorrhagic, or restenosis events during follow-up may reflect potential benefits of the stent's hybrid design and surface coating. However, given the retrospective design, small cohort size, and limited follow-up, these findings should be interpreted with caution. Larger prospective trials are warranted to confirm long-term safety and efficacy, to clarify the specific therapeutic role of coated hybrid stents in VSS, and to explore the potential for treatment under single antiplatelet therapy.
