# Erratum to “Initial experience with the antithrombogenic-coated CARESTOstent for venous sinus stenting for idiopathic intracranial hypertension: A multicenter study”

**DOI:** 10.1177/15910199261424063

**Published:** 2026-02-16

Almohammad M, Broocks G, Sporns PB, et al. Initial experience with the antithrombogenic-coated CARESTO stent for venous sinus stenting for idiopathic intracranial hypertension: A multicenter study. *Interventional Neuroradiology*. 2025. doi:10.1177/15910199251392958

In the above published article, the below-mentioned corrections have been incorporated.

- Affiliations of the authors of Christopher Nimsky and Alexander Grote have been corrected to below mentioned:
  ^9^Department of Neurosurgery, Philipps-University Marburg, Campus Marburg, Marburg, Germany
  ^10^Department of Neurosurgery, University Hospital of Giessen and Marburg, Campus Marburg, Marburg, Germany
- Affiliations of the authors of Lars Timmermann and Anja Gerstner have been corrected to below mentioned:
  ^11^Department of Neurology, Philipps-University Marburg, Campus Marburg, Marburg, Germany
  ^12^Department of Neurology, University Hospital of Giessen and Marburg, Campus Marburg, Marburg, Germany
- All the affiliations have been renumbered and updated correctly in the article.

The online version of this article has been updated.

